# Prevalence of anti-leptospiral antibodies and frequency distribution of *Leptospira* serovars in small ruminants in enzootic South Peninsular India

**DOI:** 10.14202/vetworld.2021.2023-2030

**Published:** 2021-08-06

**Authors:** Vinayagamurthy Balamurugan, Anusha Alamuri, Kirubakaran Vinod Kumar, Bibitha Varghese, Gurrappanaidu Govindaraj, Divakar Hemadri, Parimal Roy

**Affiliations:** 1 Indian Council of Agricultural Research-National Institute of Veterinary Epidemiology and Disease Informatics, Yelahanka, Bengaluru, Karnataka, India; 2 Centre for Animal Health Studies, TANUVAS, Madhavaram Milk Colony, Chennai, Tamil Nadu, India

**Keywords:** leptospirosis, sheep and goats, seroprevalence, frequency distribution, serovars, microscopic agglutination test, South Peninsular India

## Abstract

**Background and Aim:**

For understanding the epidemiology of leptospirosis, the confined abundance of several species of pathogenic leptospires and knowledge on the serovar(s) prevalent in the reservoir and carrier hosts may be a useful indicator of transmission to incidental/accidental hosts in a geographical niche. The present study was carried out to ascertain the frequency distribution of *Leptospira* serovars and the prevalence of anti-leptospiral antibodies in small ruminants (sheep and goats) in the epidemiological units (villages) in the coastal districts of enzootic regions in South Peninsular India.

**Materials and Methods:**

A total of 1167 serum samples (sheep n=299 and goats n=868) from apparently healthy animals, randomly collected from various epidemiological units were tested in microscopic agglutination test (MAT) using 18 reference *Leptospira* serovars antigens.

**Results:**

The overall seroprevalence of 40% (at 95% confidence intervals [CI]: 36.82-42.43) in small ruminants (44% [95% CI: 40.49-52.26] in sheep and 38% [95% CI: 34.96-41.41] in goats) was observed with the predominance of Icterohaemorrhagiae, Javanica, Australis, Hurstbridge, and Pyrogenes serogroup anti-leptospiral antibodies in the studied region. The Chi-squared test revealed that the presence of anti-leptospiral antibodies is significantly not independent (associated) across the administrative division (Chi-square=105.80, p<0.05) as well as for sheep (Chi-square=34.67, p<0.01) and goats (Chi-square=68.78, p<0.01). Among seropositive samples (n=462 reactors), the MAT was positive for more than one serovar in 73% of sheep (95/131) and 53% of goats (177/331), representing an overall 59% cross-reactive prevalence in small ruminants. The determined frequency distribution (varied among small ruminants) of the employed serovars representing major reactive serogroup was Icterohaemorrhagiae (29.87), Javanica (20.78), Australis (20.35), Hurstbridge (16.23), Pyrogenes (15.8), Djasmin (15.58), Bataviae (15.37), Autumnalis (14.5), Canicola (14.5), Hebdomadis (14.07), Shermani (13.64), Panama (13.42), Sejroe (12.77), etc.

**Conclusion:**

This study indicates alarmingly high seroprevalence of leptospirosis in small ruminants with existing endemicity in the studied region in South Peninsular India. Further, these prevalent serovars in the administrative division may be of use in the reference panels of antigens in the MAT in both humans and animal disease diagnostic laboratories for effective and timely diagnosis of leptospirosis and to combat the challenges in public health.

## Introduction

Small ruminants (sheep and goats) rearing is important for subsistence and economic upliftment (income generation) and social sustenance (poverty alleviation) of rural landless and marginal farmers in India. In addition, these species can adapt to a wide range of climatic and geographical conditions, including severe drought. Unfortunately, in India, like many other developing countries in the world, the true potential of this sector could not be optimally harnessed due to nutritional deficiencies and inadequate management practices, poor reproductive performance, leading to reduced productivity. In addition, the role of infectious diseases, such as brucellosis, leptospirosis, toxoplasmosis, and neosporosis, which are associated with reproductive problems/failures, may not be ignored. Among the above diseases, leptospirosis, the most neglected one, has been described as the most frequent and potentially the major infection impairing productivity in small ruminants in the endemic countries [1]. Leptospirosis is the most widely spread reemerging neglected zoonotic disease noticed in both developed and developing countries with more prevalence in tropical and subtropical rainfall regions of the world [2]. The disease incidence increases considerably during natural calamities such as cyclones and floods, causing a serious health menace to animals and humans [3]. Subclinically infected animals with host-adapted serovars serve as long-term carriers and continuous shedders of the leptospires mainly through their urine, thereby posing a risk to humans through contamination of soil and water [4] or sometimes through direct exposure. Domestic animals, which are incidental or accidental hosts, acquire infection either through a carrier or reservoir host directly or indirectly through contaminated environment sources. The affected animals show anorexia, fever, oligolactia, mastitis, icteric mucous membranes, and reproductive disorders such as abortion, infertility, stillbirths, the birth of weak calves, reduced milk yield, and productivity [5].

The leptospirosis situation in India is a cause of concern and is enzootic in all coastal states and union territories (Kerala, South Andaman, Gujarat, Maharashtra, Andhra Pradesh, Tamil Nadu, etc.) where high endemicity and prevalence were recorded both in animals and humans [4,6]. The enzootic South Peninsular region is witnessed frequent upsurges in the cases of leptospirosis, both in humans and livestock, during the monsoon season [7-10]. The environmental factors, such as the high percentage of rainfall leading to waterlogging and humidity and the presence of large paddy and sugarcane growing agricultural area coupled with the dense population of small mammals (rodents) and domestic and wildlife animals, favor the occurrence and spread of leptospirosis in these regions [11,12]. In general, the livestock has a role in maintaining *Leptospira* serovars and the prevalence study in these species would help in the management and the control of the disease [13,14]. A high level of seroprevalence among the bovine population, in most of the enzootic Indian states, has been reported, wherein antibodies against more than 20 serogroups with the frequent shift in dominant serovars have been observed over the years in those states, where frequent monitoring is being carried out [12,13,15,16]. It is disappointing those studies on the prevalence and distribution of serovars in sheep and goats are very scanty in the world, including India, except for a few isolated studies [15,17-19]. Leptospirosis in sheep and goats may present as an acute or as a subclinical infection and the affected animals may show pyrexia, conjunctivitis, jaundice, anemia, anuria, hemoglobinuria, loss of appetite, irritability, diarrhea, mastitis, haemogalactia, opaque furs, epidemic abortions, etc.; whereas, the severe forms may lead to mortality of the lambs and kids [1,5,18]. Few studies have shown that sheep and goat leptospirosis have frequently been associated with serovar Hardjo, where the animal harbors the organism in the kidney as a chronic infection [20,21]. However, mortality is often associated with incidental serovars such as Pomona, Ballum, Icterohaemorrhagiae, or Grippotyphosa [1].

It is of paramount importance to study the prevalence of anti-*Leptospira* antibodies against different serogroups from different geographical locations. It is worth mentioning that for understanding the epidemiology of leptospirosis, the confined abundance of several species of pathogenic leptospires and knowledge on the serovar(s) prevalent in the reservoir and maintenance/carrier hosts may be a useful indicator of transmission to incidental/accidental hosts such as humans and livestock species in a geographical niche. Further, information on serovars circulating in various regions around the world is always wanted in the field of leptospirosis research. In addition, the prevalent serovars in the particular region may be of use in the reference panels of antigens in the microscopic agglutination test (MAT) in both humans and animal disease diagnostic laboratories.

With the above background, the present study has been undertaken in determining the frequency distribution of predominant *Leptospira* serovars and the prevalence of anti-leptospiral antibodies against various serogroups in sheep and goats of the endemic regions in South Peninsular India.

## Materials and Methods

### Ethical approval

The manuscript does not contain animal experimental trials. No ethical clearance is required for collecting small volumes of blood samples required for seroepidemiological studies, as per the Committee for the Purpose of Control and Supervision of Experiments on Animals guidelines. Moreover, samples were collected by well-trained veterinarians concerning animal welfare regulations.

### Study period and location

The present study was conducted from July 2017 to March 2018 using the random serum samples collected from apparently healthy sheep and goats from various epidemiological units (villages) in the coastal districts of Andhra Pradesh, Kerala, Tamil Nadu, and Puducherry administrative division of the South Peninsular study region, where leptospirosis is endemic and human cases are being reported regularly [4,6,8,10,17].

### Sample size

The sample size was determined for the finite population as per Snedecor and Cochran [22] formula N=Z^2^ (p [1-p/e^2^]) using Epitool (http://epitools.ausvet.com.au/content.php?page= 1Proportion), where N=sample size, Z=95% confidence level, p=25% maximum proportion (based on published data Vihol *et al*. [23]), and e is the precision level (5%). A total sample size of 289 was determined at the disaggregated level (administrative division) level separately in the study region. However, after considering the attrition rate of 10%, the total arrived sample size was 320. In the Indian context, the village was considered as an epidemiological unit (a village consisting of a group of households that pursue similar animal husbandry and socioeconomic activities). The list of villages in each state having more than 200 small ruminants (with inclusion and exclusion criteria-as per 19^th^ Livestock Census, 2012) population (http://www.dahd.nic.in/) was shortlisted, which formed the sampling frame. Accordingly, the number of required estimated sampling epidemiological units (n=29-32) arrived and was allocated randomly to the blocks or tehsils in the coastal districts of each administrative division using Epi-calculator designed by the Indian Council of Agricultural Research-National Institute of Veterinary Epidemiology and Disease Informatics (ICAR-NIVEDI) (https://www.nivedi.res.in/Nadresv2/Epical/stratified/random_ sampling.php). In each of the selected villages, the number of animal samples was calculated by the hypergeometric distribution and the maximum samples (n=10 or 11) were determined using the epidemiological calculator.

### Serum samples

**Figure-1:**
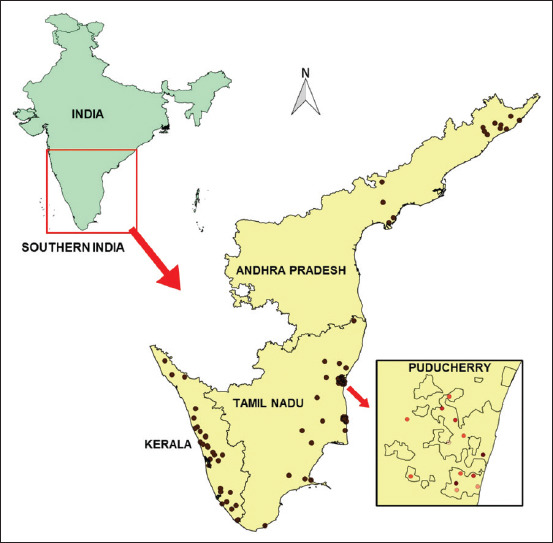
The surveyed Epi units (villages) location is depicted (as • a dot) in the GIS Map (using QGIS version 2.18.0) of the studied administrative division in the southern peninsular region of India.

In each epidemiological unit, serum samples were collected as per the sampling plan through the All India Coordinated Research Project on Animal Disease Monitoring and Surveillance (AICRP on ADMAS), a collaborating center of ICAR-NIVEDI, in the respective administrative divisions. The surveyed Epi units in the studied Andhra Pradesh, Kerala, Tamil Nadu, and Puducherry administration divisions are depicted in GIS Map (Figure-1) using QGIS Software 2.18.0 version (QGIS team, Switzerland). In the village, random blood samples of animals were collected and allowed to clot and the sera separated from the clotted blood were labeled and transported in an ice-cool container to the laboratory and the samples on receipt were stored at −80°C until further use. Since the Union Territory of Puducherry and the state of Kerala have a negligible sheep population, most/all of the sera samples were drawn from goats.

### MAT

All the sera were tested by MAT using a panel of 18 reference *Leptospira* serovars [8] covering 16 serogroups. The additional two serovars, Kaup and Copenhageni representing the Tarassovi, and Icterohaemorrhagiae serogroups, respectively, were also included in the study based on our earlier observations [8,24] of their presence. *Leptospira* live antigens required for MAT were grown in Ellinghausen, McCullough, Johnson, and Harris liquid medium. For this purpose, serovars were incubated at 29±1°C, and 5-7-day-old cultures at a concentration of 1-2×10^8^ organisms/ml were used as live antigens in MAT. The test was performed as per OIE procedures [25] and sera were tested by MAT [8,24] to determine positive reactors against different serogroups using a cutoff titer ≥100, as per the WHO/OIE recommendation [25] in endemic settings.

### Statistical analysis

The Chi-squared test was carried out in Microsoft office Excel 2016 as per the described method [8] to understand the association of *Leptospira* antibodies with a working null hypothesis (H0) of the independent presence of antibodies in sheep and goats between the species and across the studied administrative divisions.

## Results

On screening of 868 goats and 299 sheep serum samples, a prevalence of 44% (131/299) in sheep and 38% (331/868) in goats was observed with the predominance of Icterohaemorrhagiae, Javanica, Australis, Hurstbridge, and Pyrogenes, followed by Djasmin, Bataviae, Autumnalis, Canicola, Hebdomadis, Shermani, Panama, and Sejroe, serogroup anti-leptospiral antibodies in the particular areas of the studied region. The overall prevalence of 40% (462/1167) in small ruminants with a prevalence of 62.81, 41.69, 29.18, and 24.11% in Andhra Pradesh, Kerala, Tamil Nadu, and Puducherry administrative division, respectively, was observed. The details of serum samples screened and their percent seropositivity with anti-leptospiral antibodies reacted with the major serovars representing their respective serogroups in the study region of South India are presented in Tables-1 and 2. The Chi-squared test revealed that the presence of anti-leptospiral antibodies is significantly not independent (associated) across the administrative divisions (Chi-square=105.80, p<0.05) as well as for sheep (Chi-square=34.67, p<0.01) and goats (Chi-square=68.78, p<0.01).

**Table 1:** Details of sheep and goats serum samples screened and its test results by microscopic agglutination test.

Name of the states	No. of the district	No. of the Tehsil/Block	No. of the village	No. of the serum samples screened			No. of the sample positively reacted in MAT			Percentage positivity/prevalence (CI value at 95%)*		
		
				Sheep	Goats	Total	Sheep	Goats	Total	Sheep	Goats	Total
Andhra Pradesh	3	13	13	142	143	285	87	92	179	61.27 (53.06-68.88)	64.34 (56.21-71.72)	62.81 (57.1-68.22)
Kerala	8	22	29	-	319	319	-	133	133	-	41.69 (36.41-47.14)	41.69 (36.41-47.17)
Tamil Nadu	9	13	14	130	151	281	39	43	82	30.00 (22.79-38.36)	28.48 (21.89-36.14)	29.18 (24.17-34.75)
Puducherry	2	5	24	27	255	282	5	63	68	18.52 (8.18-36.7)	24.71 (19.82-30.35)	24.11 (19.49-29.43)
Total	22	53	80	299	868	1167	131	331	462	43.81 (40.49-52.26)	38.13 (34.96-41.41)	39.59 (36.82-42.43)
				Chi square value states (c^2^=105.80, p<0.05)** Sheep (c^2^=34.67, p<0.01)** Goats (c^2^=68.78, p<0.01)**						Species (c^2^=5.78 p<0.01)**		

* Parenthesis indicates – confidence interval value at 95%.

** Significance

**Table 2:** Administrative division-wise details of the prevalence of anti-leptospiral antibodies by microscopic agglutination test.

Name of the administrative division	Major prevalent anti-leptospiral antibodies against the serovars* representing their respective serogroups	Only reacted samples with *Leptospira* intermediate spp. serovars*representing serogroup# **	Percentage reactivity of the major reacted *Leptospira* serovars employed in a microscopic agglutination test
Andhra Pradesh	Ict, Heb, Bat, Aus, Sej, Dja, Tar, Aut, Jav, Can, Pan, Pyr, Hus, Pom, She, and Gri	Kau (1), Hus (3)	Cop (32.4), Heb (28.49), Bat (26.26), Aus (22.35), Har (20.67), Dja (19.55), Tar (19.55), Ban (17.32), Jav (16.76), and Can (15.08)
Kerala	Jav, She, Aus, Aut, Dja, Hus, Ict, Pan, Pyr, Can, Gri, Sej, Bat, Pom, Tar, and Heb	Kau (1), Hus (8)	Jav (21.05), She (21.05), Aus (19.55), Ban (18.8), Dja (15.79), Hus (14.29), Cop (14.29), Pan (13.53), Pyr (12.78), and Ict (10.53)
Tamil Nadu	Ict, Jav, Pan, Pyr, Hus, Aus, Dja, Can, She, Bat, Aut, Heb, Sej, Pom, Kau, and Tar	Hus (6)	Can (24.39), Jav (21.95), Hus (21.95), Aus (18.29), Pyr (18.29), Bat (13.41), Har (12.2), Pom (10.98), and She (10.98)
Puducherry	Can, Jav, Hus, Aus, Pyr, Bat, Sej, Pom, She, Ict, Gri, Heb, Dja, Aut, and Tar	Kau (2), Hus (8)	Cop (33.82), Jav (29.41), Pan (26.47), Pyr (25), Hus (23.53), Aus (19.12), Dja (17.65), Ict (16.18), Can (13.24), and She (11.76)
Total	Ict, Jav, Aus, Hus, Pyr, Dja, Bat, Aut, Can, Heb, She, Pan, Sej, Tar, Pom, and Gri	Kau (4), Hus (25)	Cop (22.51), Jav (20.78), Aus (20.35), Hus (16.23), Pyr (15.8), Dja (15.58), Bat (15.37), Ban (14.5), Can (14.5), Heb (14.07), and She (13.64)

* Aus=Australis; Aut=Autumnalis; Ban=Bankinang; Can=Canicola; Har=Hardjo; Heb=Hebdomadis; Pyr=Pyrogenes; Tar=Tarassovi; Kau=Kaup; Ict=Icterohaemorrhagiae; Cop=Copenhageni; Pom=Pomona; She=Shermani; Gri=Grippotyphosa; Hus=Hurstbridge; Jav=Javanica; Pan=Panama; Dja=Djasiman; Bat=Bataviae. ^#^Kaup and Hurstbridge representing their respective Tarassovi and Hurstbridge serogroup, respectively.

** Parenthesis indicates No. of samples reactivity

The district-wise details of seroprevalence in small ruminants in the different administrative divisions in the study region are available from the authors as supplementary tables on request. It is pertinent to mention that the 462 seropositive reactors, 73% of sheep (95/131) and 53% (178/331) of goats serum samples were positive for more than one serovar in MAT. Thus, overall 59% of small ruminant sera were reactive to more than one serovar. The cross-reactivity of sheep and goats sera with different serovars is presented in Table-3. The anti-leptospiral antibodies against major predominant serovars representing their respective serogroup were determined by the frequency distribution in different species with their overall reactivity percentage which is summarized in Table-4.

**Table 3:** Cross-reactivity among different *Leptospira* serovars in small ruminants in the South Peninsular region.

	Ban	Aus	Can	Sej	Heb	Pyr	Tar	Kau	Ict	Cop	Pom	She	Gri	Hus	Jav	Pan	Dja	Bat
Ban	-	32	23	16	19	17	7	5	7	15	6	13	6	10	12	13	9	16
Aus	-	-	25	16	44	21	10	8	10	24	10	16	8	10	16	16	17	20
Can	-	-	-	25	16	16	8	4	7	13	9	7	6	10	11	10	9	18
Sej	-	-	-	-	18	10	12	5	9	11	12	7	4	6	9	8	10	15
Heb	-	-	-	-	-	9	13	8	4	23	7	11	7	10	11	12	12	16
Pyr	-	-	-	-	-	-	10	5	9	16	6	10	6	11	16	12	10	11
Tar	-	-	-	-	-	-	-	3	2	8	5	6	3	10	3	6	9	10
Kau	-	-	-	-	-	-	-	-	3	2	5	2	3	2	1	3	5	5
Ict	-	-	-	-	-	-	-	-	-	4	5	4	5	3	6	5	6	6
Cop	-	-	-	-	-	-	-	-	-	-	9	13	9	11	30	27	32	35
Pom	-	-	-	-	-	-	-	-	-	-	-	8	6	9	8	6	8	12
She	-	-	-	-	-	-	-	-	-	-	-	-	6	9	17	11	13	9
Gri	-	-	-	-	-	-	-	-	-	-	-	-	-	9	13	10	7	6
Hus	-	-	-	-	-	-	-	-	-	-	-	-	-	-	26	14	11	9
Jav	-	-	-	-	-	-	-	-	-	-	-	-	-	-	-	28	24	14
Pan	-	-	-	-	-	-	-	-	-	-	-	-	-	-	-	-	30	12
Dja	-	-	-	-	-	-	-	-	-	-	-	-	-	-	-	-	-	11
Bat	-	-	-	-	-	-	-	-	-	-	-	-	-	-	-	-	-	-

Abbreviations are the same as mentioned in Table-2

**Table 4:** Frequency distribution of predominantly reacted *Leptospira* serovars in small ruminants.

*Leptospira* serovars	Overall reactivity (No.)			Frequency reactivity (%)		
	
	Sheep	Goats	Total	Sheep	Goats	Total
Copenhageni	69	35	104	31.42	26.72	22.51
Javanica	71	25	96	29.00	19.08	20.78
Australis	65	29	94	28.40	22.14	20.35
Hurstbridge	57	18	75	22.66	13.74	16.23
Pyrogenes	56	17	73	22.05	12.98	15.80
Djasiman	49	23	72	21.75	17.56	15.58
Bataviae	34	37	71	21.45	28.24	15.37
Bankinang	48	19	67	20.24	14.50	14.50
Canicola	36	31	67	20.24	23.66	14.50
Hebdomadis	36	29	65	19.64	22.14	14.07
Shermani	47	16	63	19.03	12.21	13.64
Panama	47	15	62	18.73	11.45	13.42
Hardjo	34	25	59	17.82	19.08	12.77
Tarassovi	21	18	39	11.78	13.74	8.44
Pomona	23	13	36	10.88	9.92	7.79
Icterohaemorrhagiae	28	6	34	10.27	4.58	7.36
Grippotyphosa	24	6	30	9.06	4.58	6.49
Kaup	14	5	19	5.74	3.82	4.11

## Discussion

The prevalence of anti-leptospiral antibodies against different serovars has been reported from different Indian states through many surveys in various animal species including humans. Further, various studies on leptospirosis in the sheep and goats over the years in different states showed various prevalent percentages with different reactive serovars, which indicate the endemicity of the disease in these regions. The observed prevalence in the study was concurrent with those of previous studies conducted in India over the years, which have shown variation in the seroprevalence of different livestock species [8,26]. The seroprevalence of 22-29% in Gujarat [18,19,23,27,28]; 9.5-47% in Tamil Nadu [17,29-31]; 27-33% in Kerala [10,32]; 23-29% in Andaman [10,15,33]; and 22-29% in different states located in various agroclimatic zones/region of India [10] in apparently healthy sheep and goats through various surveys has been reported. Further, leptospirosis from the non-endemic regions (Jammu and Kashmir, Uttarakhand, Mizoram, Punjab, Uttar Pradesh, Bihar, Rajasthan, Madhya Pradesh, and Telangana) with seropositivity of 7-15% in sheep and goats have also been reported [10].

Employing reference panel of 18 serovars representing 16 serogroups in the MAT instead of few serovars, it was evident from the study that it could detect more seroreactive animals at 1:100 titer in MAT. Further, it was observed the change in the pattern against predominant serovars such as Djasmin, Panama, Hurstbridge, and Shermani over a period. Moreover, the inclusion of serovar Copenhageni in the panel not only helped to detect an additional positive case (n=18) at 1:100 titer but also implied the need for inclusion of additional serovars from the same serogroup, particularly when information regarding the circulating serovars in a given region is limited. It is fascinating that the reactivity of field serum samples from the different regions to the different serovars representing the same serogroup to serovar Copenhageni at a cutoff titer of 1:100 did not show similar reactivity with Icterohaemorrhagiae. Nevertheless, in this study, determination of endpoint titration of the individual serovar reactivity of positive samples was not performed, as the main focus of the study was the frequency distribution of *Leptospira* serovars and its prevalence of anti-leptospiral antibodies; therefore, the variation in the titer of the sample could not be observed on the reactivity of different serovars representing the same serogroup. The other study by employing three serovars (Copenhageni, Icterohaemorrhagiae, and Lai) representing the Icterohaemorrhagiae serogroup and two serovars (Kaup and Tarassovi) representing Tarassovi serogroup in MAT with endpoint titration of cattle serum samples from different geographical locations of India revealed that there was 1-2-fold titer variation on the reactivity of different homologous serovars within specific serogroup (unpublished data). A similar observation was also earlier reported from other endemic countries [34].

The high seropositivity observed in Andhra Pradesh could suggest that in some cases cross-reactions with different serovars but not the false-positive results, as the MAT employed was the gold standard, which is highly specific for the detection of anti-leptospiral antibodies for the diagnosis of leptospirosis. In endemic settings, though we found high cross-reactivity with multiple serovars, the higher specific antibodies titer (≥1:100) to anyone serovar may indicate possible recent infection with that serovar or can be concluded as past infections or multiple infections [35]. In the above context, it would be ideal to carry out endpoint titration against each serovar to obtain more conclusive results. However, the pattern of reactivity of small ruminant serum observed in the study was similar to that observed in the earlier study conducted by Balakrishnan *et al*. [36], in cattle and humans. The only exception was that this study did not cover Ballum serogroup, while minimal reactivity (14.5%) was observed against Bankinang serovar representing Autumnalis serogroup. Whereas, serovars Copenhageni (22.5%)/Icterohaemorrhagiae (7.36%) and Australis (20.35%) representing Icterohaemorrhagiae (30%) and Australis (20%) serogroups were the additional predominant, besides the major reactive Javanica (20%), Hurstbridge (16%), and Pyrogenes (16%) serogroup anti-leptospiral antibodies. Further, the observed cross-reactions were mainly between the serovars Australis, Copenhageni, Hebdomadis, Bataviae, Tarassovi, and Javanica followed by Canicola and Bankinang. Among various serovars, Australis showed the highest cross-reactivity, followed by Copenhageni, Javanica, Hebdomadis, Bankinang, and Bataviae. Overall, the inclusion of Panama, Copenhageni, and Djasmin serovars in the panel as compared to the earlier studies [10,15,17,30] increased seropositivity to the tune of 11% (49/462). This indicates the importance of the inclusion of additional serovars in the MAT panel to identify the changing trend of prevalent serovars and also to identify if any emerging or reemerging serovars in different livestock species in the various geographical niche over the years.

The Chi-squared test implies the existence of similar agro-ecological, geographical factors, and other animal management practices prevailing in the region and associated risk factors in the sampled areas that might contribute to the leptospiral occurrence, which is concurrent with earlier reports [16,35-37]. The predominance of one/more serovars over the others and the change in those trends over the years in a particular geographical region and the role of livestock species in maintaining several predominant serovars has been well documented [12,13,15]. The epidemiological importance of the observed anti-leptospiral antibodies of serogroups in the particular areas, its source of transmission, the role of small ruminants as maintenance or accidental host and spreaders, the impact of small ruminants health and production including human health are to be studied further, as prevalence variation was observed within different areas in the studied administrative division. This observed variation may be due to the prevalence of different serovars in that particular environmental condition. In addition, the contribution of acute water stagnation as a result of heavy rainfalls/rains leading to the spread of leptospiral pathogens and the resultant change in leptospiral predominance pattern has also been reported [14]. Further, the low positivity for Hardjo serovar in the two states might be due to less population of maintenance host (sheep), which may harbor serovars Hardjo and Pomona [38] organism in the kidney as a chronic infection [20,21]. Nevertheless, mortality is often associated with incidental serovars such as Pomona, Ballum, Icterohaemorrhagiae, or Grippotyphosa [1]. Moreover, in Gujarat state, the reported anti-leptospiral antibodies predominantly against serogroups of Hardjo, Canicola, Pomona, Pyrogenes, Bankinang, Grippotyphosa, and Australis in clinically ailing goats [28] indicate the presence of carrier state and possible role in disease transmission [23]. The most important maintenance hosts are small mammals (rodents), which may transfer the infection to domestic farm animals, dogs, and humans and the extent of transmission depends on various epidemiological factors, including climate, population density, and the degree of contact between maintenance and accidental hosts [39]. Rats are generally maintenance hosts for serovars of the serogroups Icterohaemorrhagiae, whereas dairy cattle as maintenance host may harbor serovars Hardjo, Pomona, and Grippotyphosa; pigs may harbor Pomona, Tarassovi, or Bratislava; and dogs may harbor serovar Canicola [38]. Therefore, the observed high positivity for serovars Copenhageni, Icterohaemorrhagiae, Javanica, and Australis could be due to the presence of seropositivity hosts and its possible contact with small ruminants in the studied regions, as the various percentage of the seroprevalence in different livestock species have been reported [8,26].

This study describes the prevalence of anti-leptospiral antibodies against various *Leptospira* serogroups and frequency distribution of the predominant serovars in the small ruminants in endemic South Peninsular India with certain limitations, such as host factors such as age and sex, was not available for multifactorial analysis, and the target of 320 samples envisaged for each state, could not be achieved due to some administrative issues. This study not only indicates the antibodies reactive to the emerging pathogens of different serovars, the change in the trend of predominant serovars prevalent over a period [12,13,15] but also shows the high prevalence of antibodies against intermediate serovars as well as cross-reactivity with other serovars. Therefore, the information regarding the detailed distribution of serovars would help in providing an early and accurate diagnosis so that rapid and appropriate treatment could be undertaken to reduce the extent of the problem associated with the disease in humans.

## Conclusion

The study has shown the predominance of Icterohaemorrhagiae, Javanica, Australis, Hurstbridge, and Pyrogenes, followed by Djasmin, Bataviae, Autumnalis, Canicola, Hebdomadis, Shermani, Panama, and Sejroe, serogroup antibodies along with previously observed anti-leptospiral antibodies against different serovars covering different *Leptospira* species in the particular areas of the studied region. The above prevalent serovars in sheep and goats along with the major prevalent serovars in other livestock species in the particular region may be of use in the reference panels of *Leptospira* antigens in MAT in both humans and animal disease diagnostic laboratories for providing an accurate diagnosis for leptospirosis. Further, the understanding of the epidemiology of leptospirosis would help in achieving adequate surveillance and accurate diagnosis, which, in turn, helps in planning and alleviating the leptospirosis burden in endemic regions. These findings may guide public health specialists, researchers, and policy-makers to implement appropriate control measures and help to reduce the impact and challenges of zoonosis in the One Health approach.

## Authors’ Contributions

VB: Designed and conceptualized the work with overall monitoring, analyzed and interpreted the data, and wrote the original draft and edited the manuscript. AA, KVK, and BV: Carried out laboratory experiments. KVK and GG: Analyzed the data and edited the manuscript. DH: Provided serum samples and edited the manuscript. PR: Provided guidance and support to carry out the research work. All authors read and approved the final manuscript.
